# Supplementary figures and images for: Rhizoctonia solani disease suppression: addition of keratin-rich soil amendment leads to functional shifts in soil microbial communities
Source: FEMS Microbiol Ecol. 2024 Mar 18;100(4):fiae024. doi: 10.1093/femsec/fiae024 (PMC10959553; doi:10.1093/femsec/fiae024)

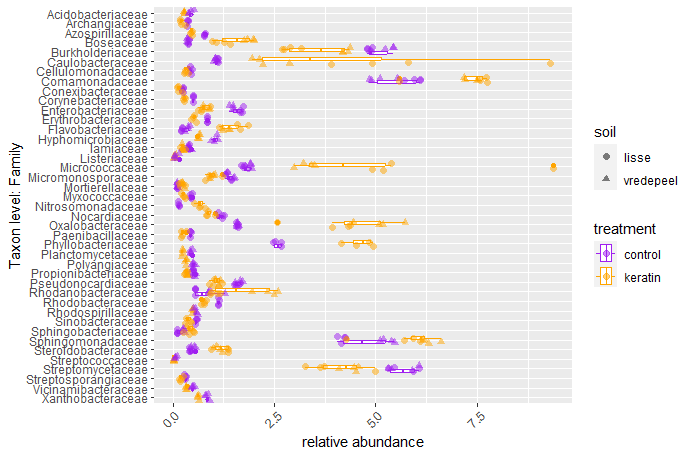

Supplement: fiae024_Supplemental_Files [file fiae024_supplemental_files.zip › Supplementary_Fig1.png]

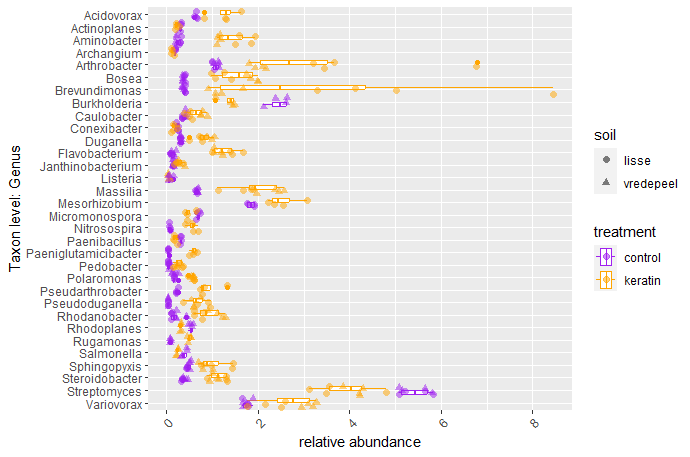

Supplement: fiae024_Supplemental_Files [file fiae024_supplemental_files.zip › Supplementary_Fig2.png]
